# Deep eutectic solvents as an emerging platform for microbial carotenoids extraction: State of the art, prospects, and challenges

**DOI:** 10.1016/j.fochx.2025.103368

**Published:** 2025-12-07

**Authors:** Gul Muhammad, Zhenglong Li, Cheng-Ye Ma, Qiong Wang, Ximing Zhang

**Affiliations:** a College of Biosystems Engineering and Food Science, Zhejiang University, Hangzhou 310058, China; b Institute of Zhejiang University-Quzhou, Quzhou 324000, China; c National Key Laboratory of Biobased Transportation Fuel Technology, Zhejiang University, Hangzhou 310058, China

**Keywords:** Natural carotenoids, Microbial carotenoids, Deep eutectic solvents, Biomass pretreatment, Carotenoid extraction

## Abstract

Microbial carotenoids are promising alternatives to synthetic ones in food, cosmetics, nutraceuticals, and pharmaceuticals, offering antioxidant, anti-aging, and anti-cancer benefits. However, the conventional extraction methods commonly used for carotenoids often rely on petrochemical solvents, raising environmental and health concerns. In this context, deep eutectic solvents (DESs) have emerged as sustainable alternatives due to their low toxicity, cost-effectiveness, ease of preparation, biodegradability, recyclability, and excellent compatibility. This review discusses the history, classification, toxicity, and biodegradability of DESs, along with recent advances in microbial carotenoid recovery, focusing on pretreatment, extraction, and underlying mechanisms, followed by the discussions on sustainability assessment and current technological challenges. While DES-based processes show significant potential within green chemistry, their feasibility for large-scale, commercial applications remains to be validated. By providing a critical overview, this study aims to guide further research and development in eco-friendly downstream processing of microbial carotenoids, bridging the gap between scientific investigation and industrial practice.

## Highlights

• Deep eutectic solvents are emerging sustainable solvents for the extraction of carotenoids.
• Microbial biomass pretreatment and extraction of carotenoids using deep eutectic solvents have been reviewed.
• The mechanism of pretreatment and extraction are comprehensively discussed.
• Sustainability assessment, technological challenges and future perspectives are proposed.

## Graphical abstract

**Figure:**
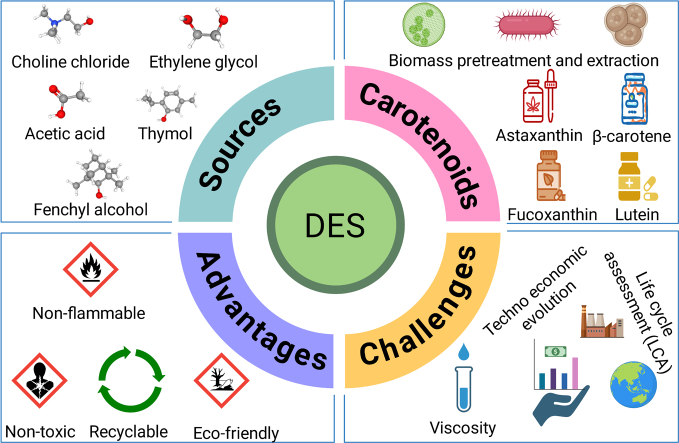

## 1 Introduction

Carotenoids (tetraterpenoids) are fat-soluble pigments having a structure that consists of eight molecules of 5‑carbon isoprenoid. Carotenoids contain 40‑carbon atoms, and two terminal rings that determine their color (Ashokkumar et al., 2023). Around 600 carotenoids are found in nature; 40 of those are consumed in the human diet and categorized into oxygen-containing carotenoids (xanthophylls) such as astaxanthin, lutein, and zeaxanthin, and hydrocarbon carotenes including α-carotene, β-carotene, and lycopene (Cheng et al., 2020). Plants are the conventional source of natural carotenoids, while microbes, specially microalgae is becoming more famous as a source (Park et al., 2022).

Carotenoids are important for many biological functions and have important applications in nutraceuticals, cosmetics, pharmaceuticals, and food supplements due to their antioxidant, anti-aging, and anti-cancer activities (Huang et al., 2025). Carotenoids have many medicinal characteristics that are often used as preventives against diseases (cataract, cancer, and diabetes) (Sathasivam & Ki, 2018). There has been growing market demand for carotenoids, with their global market value increasing from US$ 1.45 billion in 2024, and is anticipated to grow with a compound annual growth rate (CAGR) of 5.4 %, reaching a market value of US$ 2.45 billion by 2034 (Mussagy, Caicedo-Paz, et al., 2024; Mussagy, Hucke, et al., 2024). Therefore, the development of next-generation, naturally synthesized microbial pigments is important, specially lutein, astaxanthin, and β-carotene combined to gather accounts approximately 60 % of the total market value (Ashokkumar et al., 2023).

The most widely used method for carotenoid extraction is solvent extraction using organic solvents (*e.g.*, hexane, and isopropanol) (Saini & Keum, 2018). Conventional approaches are time-consuming as they involve multiple steps with extended extraction times, thereby hindering their feasibility. The efficiency of these methods is restricted by the excessive amounts of solvents needed and the possibility of incomplete extraction of the carotenoids. In addition, the health and safety issues related to organic solvents are a great risk factor, which requires strict safety measures to be taken to prevent workers endangerment (Morón-Ortiz et al., 2024). These limitations have motivated the researcher to seek more viable and effective extraction approaches. In this regard, deep eutectic solvents (DESs) have emerged as an innovative approach that aligns with the principle of green chemistry. DESs are easy to synthesize as they involve only mixing of a hydrogen bond acceptor (HBA) with a hydrogen bond donor (HBD). These materials are inexpensive to synthesize, can be prepared from biodegradable species, which produces a more environmentally friendly and lower-cost solvent (Liu et al., 2021; Spittle et al., 2022). DESs offer a wide range of potential uses in various fields, such as in the metal processing industry, biofuel production, extraction of compounds from plants (Alam et al., 2021) and microbes (Joshi et al., 2023; Mussagy et al., 2025; Sportiello et al., 2024). Recently, the use of DES for microbial biomass processing has gotten more attention for pretreatment and extraction of high value-added compounds. Mostly, hydrophilic DESs (HPLDESs) are used for microbial biomass pretreatment and the extraction of phenolic compounds (Muhammad, Xu, et al., 2024; Zainal-Abidin et al., 2024). Moreover, the progress of hydrophobic DESs (HDESs) have increased for the recovery of the hydrophobic compounds such as carotenoids (Sportiello et al., 2024). It appears that DESs can be tailored as per specific task and selective in order to isolate an interested compound by carefully selecting their constituents.

This review aims to explore the use of DESs for carotenoids recovery from microbes, by investigating their ability to improve the recovery efficiency, minimizing environmental impacts. It systematically evaluates the application of DESs for pretreatment, extraction of carotenoids, including their mechanism, which will provide a useful information for future studies. Finally, it addresses the sustainability assessment, current technological challenges (scaleup, DES recycling and recovery, product isolation), and future perspectives that are associated with its commercial applications, emphasizing the potential to revolutionize sustainable natural carotenoids production.

## 2 Methodology used for assessing the scientific output

This review presents the latest advancement in the extraction of carotenoids from microbial biomass using DESs. A comprehensive survey of scientific literature was conducted using suitable keywords and several scientific databases, such as Web of Science, Science Direct, Google Scholar, to identify the knowledge gaps and progress in this research field. In-depth search was performed using the keywords [“carotenoids” AND (“deep eutectic solvents” or “natural deep eutectic solvents” or “ eutectic mixture”) AND (“microorganisms” OR “microalgae” OR “bacteria” OR “yeast”) AND “extraction”]. All the keywords were used individually as well as in combinations to cross-check the acquired data set. Articles in this review were selected based on filtered titles and their related abstracts. More than 64 papers were published regarding the use of DESs in microbial biomass field from 2016 to 2025. Further refinement led to 24 articles, only the most suitable and relevant articles were selected while drafting the review. Approximately 95 % of the 24 papers were published in the last five years, showing the growing interest of the scientific community in the use of DESs for carotenoids extraction from microbial biomass.

## 3 Microbial biomass as a natural source of carotenoids

Several phototrophic and non-phototrophic organisms, such as archaea, bacteria, eubacteria (cyanobacteria), and eukaryotes (such as algae, fungi, and plants), are rich sources of natural carotenoids. Humans are not able to synthesize carotenoids and must obtain them from their diet (Sharma et al., 2024). Carotenoids can be categorized into two groups: (i) xanthophylls - They have different functional groups showing the involvement of oxidation in their formation. For example: keto (astaxanthin and canthaxanthin), epoxy (fucoxanthin, neoxanthin, and violaxanthin), and hydroxy groups (lutein and zeaxanthin) functional groups. (ii) carotenes- They have a simple structure composed of hydrocarbons. Lycopene is the simplest carotene; α and β-carotene are derived through end chain cyclisation (Joshi et al., 2023). Carotenoids are also found in fruits and vegetables; they are fat-soluble, and their absorption mainly depends on whether they are prepared *via* fats or oils (Sanlier et al., 2024). Furthermore, among the carotenoids, β-carotene has the highest provitamin A activity, and is present mainly in orange-color fruits, green leafy vegetables, with darker colors indicating higher contents (Singh, 2023).

In terrestrial plants, carotenoids like lutein, zeaxanthin, capsanthin, violaxanthin, and neoxanthin are in yellow and red color belonging to the xanthophyll cycle, and play a photoprotective role. Marigold flowers contain high amount of lutein (Rodriguez-Amaya, 2019). Green vegetables, including spinach, kale, broccoli, and lettuce, are also abundant sources of lutein, zeaxanthin, and β-carotene. Additionally, tropical fruits, in particular mango, papaya, peaches, squash, and oranges, also contain a high amount of lutein and other carotenoids. Also, microalgae are an important source of carotenoids, in addition to other compounds such as lipids, polyunsaturated fatty acids, proteins, and vitamins. Algae are classified into green (Chlorophyta *i.e. Chlorella, Dunaliella*), brown (Phaeophyta*i.e. Laminaria and Saccharina*), and red algae (Rhodophyta i.e., *Porphyridium* and *Gracilaria*), depending on their primary photosynthetic pigments (Generalić Mekinić et al., 2023; Manam, 2024; Rodriguez-Amaya, 2019). Microalgae produce several commercially relevant carotenoids, including astaxanthin, β-carotene, canthaxanthin, lutein, and lycopene, which have antioxidant properties. For example, brown seaweeds like *Laminaria japonica* and *Undaria pinnatifida* are abundant in fucoxanthin (Anjana & Arunkumar, 2024), while microalgae such as *Haematococcus pluvialis, Chlorella zofingiensis, C. protothecoides,* and *C. ellipsoidea* are the primary sources of astaxanthin, lutein, and zeaxanthin (Cezare-Gomes et al., 2019).

Similar to microalgae, some fungal species produce significant amounts of carotenoids that accumulate intracellularly. Yeast has the ability to grow on high sugar media, which makes it an industrially viable source of carotenoids. Various species of yeast, including *Xanthophyllomyces dendrorhous*, *Rhodotorula glutinis*, and *Phaffia,* which are being explored to maximize the carotenoids production for commercial applications (Naz et al., 2023). One well-known example is the naturally occurring orange-red carotenoid canthaxanthin in the *Cantharellus cinnabarinus*. Moreover, fungal groups such as *Zygomycetes*, *Ascomycetes,* and *Basidiomycetes* show higher carotenoid production relative to other organisms, which can be beneficial for industrial applications (Langi et al., 2018).

## 4 DESs as a sustainable source for carotenoids recovery

### 4.1 Historical background of DESs

**Fig. 1:**
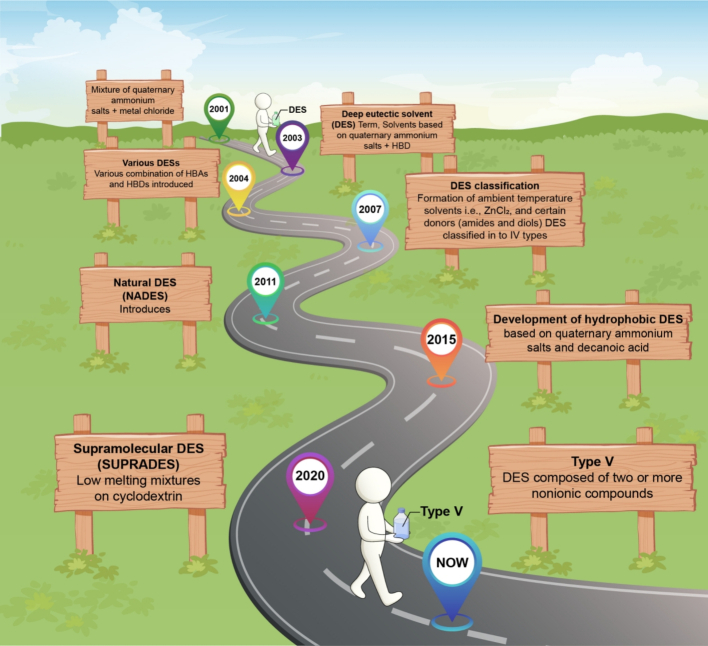
Landmarks and milestones in DES research.

DESs and ILs have been discussed in the literature over the years. DESs are considered as of great interest over ILs due to their eco-friendliness. ILs are still experiencing poor biodegradability and toxicity (de Jesus & Maciel Filho, 2022; Oyoun et al., 2023). DESs were introduced in early 2000 for eutectic solvents (ESs) (Abbott et al., 2001). Abbott et al. (2001) investigated a study on ESs by mixing quaternary salts [Y(CH_2_)_2_N (CH_3_)_3_] Cl, Y = OH or Cl) with ZnCl_2_ or SnCl_2_. After that, in 2003, Abbott et al. (2003) proposed a fascinating category of nonaqueous solvents known as DESs. These solvents are characterized by eutectic mixtures of ammonium salts, such as choline chloride (ChCl), which acts as an HBA, combined with HBD like urea. Since their inception, research on DESs has increased significantly, with a variety of HBAs, including alditols, amino acids, and aromatic or aliphatic acids, *etc.*, which can be mixed with quaternary salts. Over the years, DESs have evolved to incorporate hydrated transition metal halides or nitrates with organic ligands as HBDs. In recent years, innovations have emerged using non-ionic HBAs and HBDs, like terpenoids, highlighting the ongoing development of DES formulations, as shown in Fig. 1. One significant feature of DES is that their customizable properties, which are tailored according to their constituents, make them versatile for various scientific applications. Due to their potential, DESs have received relatively more attention in the microbial field for biomass processing and the production of valuable compounds. DESs offer unique characteristics that position them as promising substitutes to traditional volatile organic solvents, and scientists globally are rapidly exploring the full potential of DESs in microbial biomass processing.

### 4.2 Classification of DESs

**Fig. 2:**
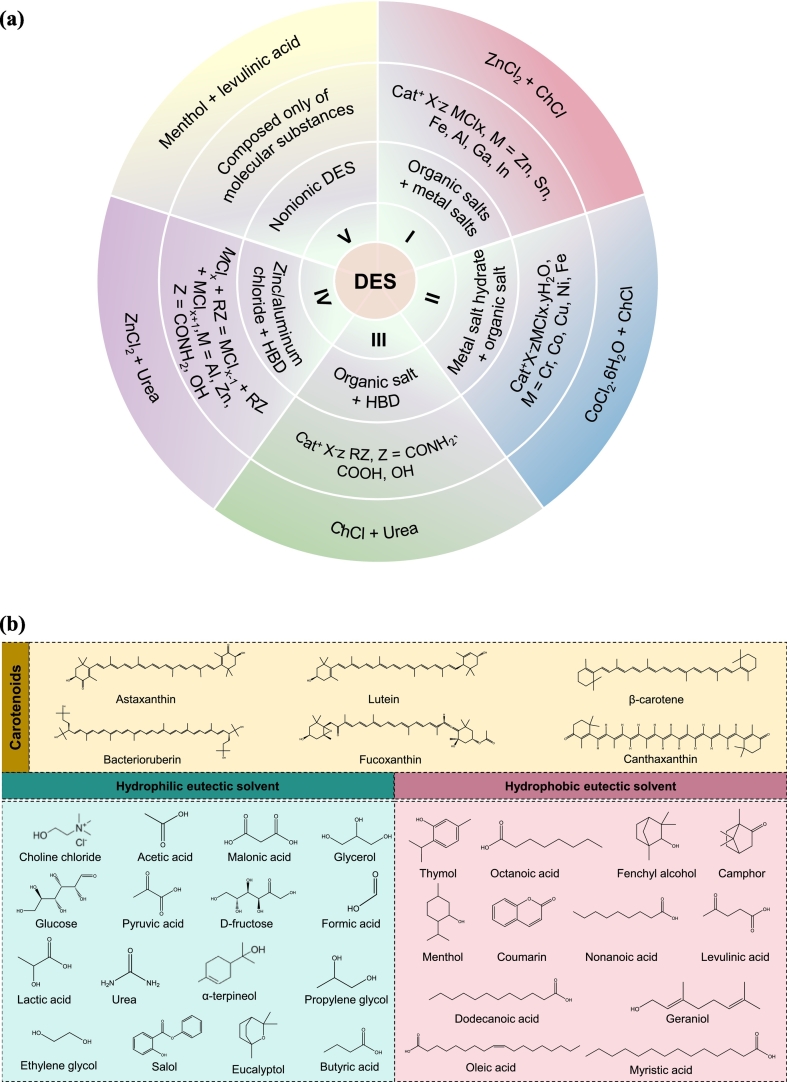
Types of DESs with examples (a); common components used to prepare DES in microbial biomass processing for carotenoids (b).

DES refers to the state of liquid when polar components (solid or liquid) are mixed *via* H-bonding at room temperature. Different combinations of individual components, various sources, and physicochemical properties have been investigated and classified into multiple categories (Chevé-Kools et al., 2025; Hansen et al., 2021; Abbott et al., 2003). DESs can also be divided on the basis of general formula Cat^+^ X^−^zY, where Cat^+^ (ammonium, phosphonium, or sulfonium cation) represents a cationic moiety, X represents Lewis base (mostly a halide anion), Y refers to a Brønsted or Lewis acid, and z for a number of Y molecules interacting with the related anion (Muhammad, Xu, et al., 2024). On the basis of the acid and base, DESs have been categorized into five classes, as shown in Fig. 2**(a)**. Type I includes quaternary ammonium salt (QAS) with a metal chloride, while Type II is a mixture of QASs with metal halides that have a lower melting point than Type I, because of the presence of water of hydration. These types of DESs are preferable in commercial operations because they are more economically viable and moisture-resistant than Type I. Whereas Type III is a mixture of QASs with HBDs, like alcohols, amides, and carboxylic acids. Type III DESs are versatile because of the availability of various HBDs and several are biodegradable (Smith et al., 2014). Type IV is a mixture of metal chloride and HBDs, and is considered as an emerging class of DESs. Type V DES is a new class of DES, which is a mixture of non-ionic HBDs and HBAs where a substantial H-bonding network contributes to their distinctive properties (Omar & Sadeghi, 2023). Most commonly used components for preparing HPLDESs and HDESs for the pretreatment and extraction of carotenoids are illustrated in Fig. 2(b).

#### 4.2.1 Natural deep eutectic solvents (NADESs)

NADESs are a subclass of DESs include five types: ionic (acid and base), neutral (sugars or sugars combined with polyalcohols), neutral with acids (sugars or polyalcohols combined with organic acids), neutral with bases (sugars or polyalcohols combined with organic bases), and amino acid-containing NADESs (formulated from amino acids and sugars or organic acids) (González et al., 2018). NADESs are recognized for their environmental friendliness, sustainability, and safety compared to traditional solvents (Socas-Rodríguez et al., 2021). Generally, ChCl, QAS, are used as HBAs; other HBAs include alcohol, fatty acid, and organic ammonium (Zhang et al., 2023). Sometimes, particularly fatty acids serve as HBAs and HBDs (Freitas et al., 2024). NADESs can be synthesized through conventional heating and stirring or by mechanically grinding the components together to produce a transparent liquid. Another approach is freeze drying, in which e the resulting mixture is mixed with water before freeze drying (Kaoui et al., 2023). Apart from the simplicity of NADESs preparation, their properties are tailorable because of the diverse range of components (HBA and HBD) which can be exploited (Van Osch et al., 2019). Furthermore, on the basis of their composition, NADESs can outperform traditional solvents in terms of yields for compounds that are polar or non-polar (carbohydrates, carotenoids, phenols, and protein) and other lipid-soluble components extracted from various sources (blueberry leaves, pomegranate peel, pumpkin, and rosemary) (Ji & Brooks, 2025). The efficacy of NADESs for biomolecules extraction can be credited to their extensive H-bonding (Ristivojević et al., 2024). Recent review articles have highlighted the potential of NADESs/DESs as a sustainable method for extracting high value-added compounds from agricultural and marine biomass (Airouyuwa et al., 2024; Mavai et al., 2024).

#### 4.2.2 Hydrophilic and hydrophobic DESs

HPLDESs are mainly composed of hydrophilic components derived from renewable sources, including acids, alcohols, organic salts, and sugars (Rahman et al., 2025). HPLDES offers significant advantages in extracting hydrophilic compounds from the biomass. For instance, HPLDESs have been used for the recovery of phenolic compounds from various biomass sources (Alam et al., 2021; Liu et al., 2025) and polysaccharides (Ji & Brooks, 2025). On the other hand, to HPLDESs, HDESs are less common because of the insufficient availability of low-cost hydrophobic salts and other elements that can produce ESs at ambient temperature. The hydrophobicity of DESs is mainly related to the hydrophobicity of their precursor components. Two different categories of HDESs exist. The predominant studied HDESs are primarily composed of QAS with long alkyl chains such as (tetraoctylammonium bromide, methyltrioctylammonium bromide, *etc.*) (Van Osch et al., 2015). The second category consists of a combination of two neutral hydrophobic natural components; one specifically acts as HBA and the other as hydrophobic HBD, like carboxylic acids or long-chain alcohols. Ribeiro et al. (2015), introduced the first neutral-based HDESs by mixing DL-menthol (Men) with various natural carboxylic acids (CarA). A few examples related to other neutral-based HDESs are (Ter):CarA, L-(Men):CarA, and thymol (Thy):CarA (Zainal-Abidin et al., 2021). Despite their potential, only a few studies have focused on the structures of neutral-based HDESs compared to those of salt-based HDESs (Percevault et al., 2020; Zainal-Abidin et al., 2021).

### 4.3 Toxicity, and biodegradability of DESs

The toxicity of DES in carotenoid extraction is of great concern. DESs are generally considered non-toxic due to their components are derived from natural sources and fall into the subclass of DESs named as NADESs. There is an ongoing debate on their potential toxicity (Radošević et al., 2018). Various factors that affect the toxicity of DESs include molar ratio, viscosity, and the source of intermediates (Sharma et al., 2024). Therefore, the next section will address the toxicity of DESs and NADESs, as well as their biodegradability.

Generally, ESs are used for carotenoid recovery, mainly based on ChCl and terpenes. Ch is a main precursor in the synthesis of the cell membrane, and its salt form, ChCl, is considered as safe (Martínez et al., 2022). Terpenes are plant-derived compounds and so are Generally Recognized As Safe (GRAS). When NADESs are prepared using other natural compounds like sugars (Glu, Fru), sugar alcohols [*e.g.*, xylitol (Xyl), maltitol], organic acids (*e.g.*, MA, OA), and amides (U), their toxicity can vary because of the intermolecular interactions (Rodrigues et al., 2020). These interactions can significantly influence the physicochemical properties of the compounds, thereby affecting their toxicity. Radošević et al. (2018) studied the toxicity of sugar-based and non-sugar-based NADESs through HeLa and MCF-7 cell lines. The sugar-based HBDs (Glu and Fru), and non-sugar-based HBDs (U, OA, Xyl, Sorb, and Gly) were selected for the study. The findings indicated that sugar-based NADESs showed no cytotoxic effects on normal and tumour cells, further confirming their low toxicity. The presence of sugar in the NADESs, facilitated metabolism providing energy for metabolic pathways, and supporting the integrity of carbohydrates necessary for cell growth. Similar findings were observed when sugar-based HBDs were combined with betaine and proline. The inclusion of betaine and proline as osmoprotectants, reduced the detrimental effects of high osmolarity on cell development, thereby enhancing cell growth and restoring metabolic profiles. Results indicated that the NADESs having OA showed substantial toxicity on normal cell lines as well as on tumour, attributed to the formulation of toxic substances namely calcium oxalate crystals (Hayyan et al., 2016; Radošević et al., 2018). The toxicity of NADESs is linked to charge delocalization from HBDs formation. Therefore, by varying molar ratios directly influences their toxicity (Fuad et al., 2021). For instance, ChCl:malic acid (MalA) (1:1) and (1.5:1) when employed to catfish ovary cells, cytotoxicity increased as the acid content increased. This highlights the significance of varying molar ratios in toxicity assessment (Radošević et al., 2016). Hayyan et al. (2016) addressed a relationship between viscosity and toxicity, noting that NADESs having low viscosity, such as ChCl:Gly demonstrated the lowest toxicity, whereas NADESs having more viscosity, like ChCl:MalA exhibited higher toxicity. Additionally, terpene-based ESs employed for carotenoid extraction displayed different levels of cytotoxicity against Caco-2 cells. Terpenes [perillyl alcohol, camphor (Cam), Men, eucalyptol (Euc)] and myristic acid (MyrA) displayed EC_50_ range of 0.5 to 1.1 mg/mL against Caco-2 cells. Likewise, extracts from shrimp shells, mussels, and *H. pluvialis* with Men:MyrA exhibited EC_50_ of 1.5, 1.8, and 3.3 mg/mL against Caco-2 cells, respectively (Rodrigues et al., 2020).

Few studies have been carried out on the biodegradation of DESs. The biodegradability of NADESs can be assessed using a closed-bottle test, as per guidelines set by Organization for Economic Co-operation and Development (OECD). According to these guidelines, a substance is classified as “readily biodegradable” if the removal efficiency of a substance is 60 % of theoretical oxygen demand in 10 days throughout 28 days period (Koh et al., 2023). In this regard, Huang et al. (2017) examined the biodegradability of NADESs where ChCl and Gly combined with amino acids, alcohols, and sugars, achieved over 70 % biodegradability after 28 days, thus rated all tested NADESs as “readily biodegradable”. Additionally, Radosevic et al. (2015) reported the biodegradability of ChCl-based DES with Glu, Gly, and OA, achieving >60 % biodegradability efficiency, referring to them as “readily biodegradable”. ChCl:Gly showed the highest biodegradation level (96 %), while ChCl:OA showed the lowest (68 %) level of biodegradation. In summary, the aforementioned studies have shown that ESs exhibit lower toxicity levels in both aquatic and terrestrial systems up to certain levels. Although ESs also exhibited significant biodegradability, but they could not be labelled as readily biodegradable. Most of the aforementioned studies ChCl as a HBA, has been used, highlighting the need for further research on the biodegradability of DES involving different HBAs.

### 4.4 Computational tools for selection of DESs

In recent years, computational tools for example, Conductor-like Screening Model for Realistic Solvents (COSMO-RS), have been employed to screen solvents for the recovery of biomolecules, as this model requires only molecular information about the system. These tools significantly reduce the time and cost associated with traditional screening methods for DESs, which are time consuming and expensive. Furthermore, they simulate the solubility of desired compounds in DES, thereby predicting their potential for extraction and separation. Additionally, COSMO-RS can be employed to simulate viscosity, density, and biological toxicity. A minimal deviation between the ideal DES simulated by the software and experimental findings indicates the high validity of the DES for practical usage (Ma et al., 2025).

The COSMO-RS model was employed to simulate the evaluation of twenty-four HPLDESs and HPDESs to extract fucoxanthin from *Tisochrysis lutea* algae. When Thy and tetrabutylammonium chloride were examined as HBA, an enhancement in fucoxanthin extraction was observed; conversely, the use of Men as HBA resulted in an opposing effect. Among the five HBAs examined, Thy yielded the best results, which aligns with the experimental results (Xu et al., 2022). Viñas-Ospino, Panić, et al. (2023) employed COSMO-RS to screen out sixty-eight HPLDES and HPDES for the extraction of carotenoids (β-carotene and β-cryptoxanthin) from orange peel. Due to the high viscosity of HPLDES, 30 % water used to reduce their viscosity. The findings of the study indicated that the HPLDES containing ChCl as HBA, U, Sorb, and sucrose as HBDs were ineffective in solubilizing both carotenoids. In contrast, the HPDES using Men as HBA and Euc and Cam as HBD showed the best results, which were validated by the experimental and predicted results.

Another method is molecular dynamics simulations (MD simulations), which have become a prominent approach for studying the nanostructures and dynamic characteristics of materials. MD simulations provide direct insights related to molecular processes and can elucidate and predict the mechanism of molecular interactions (Tolmachev et al., 2022). Recently, a study carried out by Muhammad, Jahanbakhsh-Bonab, et al. (2024) to evaluate the properties (viscosity and density) of HPLDES [ChCl:Gly (1:2), ChCl:Eg (1:2 and 1:3)], and HPDES [Men:LauA (2:1), Men:AA (1:1, 1:2, and 1:3)] for microbial biomass pretreatment and results compared with the experimental outcomes. The simulated viscosities and densities were consistent with the experimental data, exhibiting a deviation ranging from 0.85 % to 15.8 %. In the same study, the dissolution of cellulose *via* ChCl:Eg (1:2) was also investigated through simulation to gain deeper insights at the nano-scale level. Furthermore, the screening of solvents [water, ethyl acetate, 2-methyltetrahydrofuran (2-MTHF), and ethanol] and the mechanism of lutein extraction were also explored, specifically to enhance understanding of interaction, diffusion coefficients, and the study of HBDs formation between lutein and the solvents, using MD simulations. Moreover, in another study, a Density Functional Theory (DFT) based approach was applied to elucidate the interaction between β-carotene and Thy-Men DES, which can help to understand interactions and serve as an indicator for stability in developing solvent technology and enhancing extraction methodologies (Ojeda et al., 2025).

Recently, Bragagnolo et al. (2025) published a comprehensive review on the application of computational tools, suggesting that solvent validation should not solely concentrate on the top-ranking solvents. To strengthen the short-listed solvents selection process under experimental conditions is also needed to prevent the premature discarding of promising candidates, thereby enhancing the accuracy of solvent selection. Despite of that the only few studies on the application of computational tools in the field of microbial biomass are available, as the afore mentioned tools have shown promising results in exploring and screening the DESs for carotenoids recovery.

### 4.5 Mechanism of microbial biomass pretreatment with DESs for carotenoid extraction

Currently, carotenoids are extracted using petrochemical origin solvents (*i.e.*, hexane and acetone), which pose environmental and health risks because of the production of toxic volatile compounds. In this case, these traditional solvents are appealing alternatives. Therefore, in response to these concerns, green chemistry promotes the use of sustainable and efficient solvents for carotenoid recovery. In this regard, tailor-made solvents (DESs) offer several advantages, *i.e.*, lower environmental impacts, a simple synthesis method, and less toxic. Specially when natural components are employed, these solvents are named as NADESs (Benvenutti et al., 2019). Some of them are used in the food and pharmaceutical industry. NADESs have the capability to stabilize carotenoids as these compounds are sensitive or being directly applied in cosmetics, food supplements, nutraceuticals, or pharmaceutical products (Yu et al., 2022). The high viscosity of NADESs and their molecular interactions with carotenoids probably contribute to their protective effects (Yu et al., 2022).

**Fig. 3:**
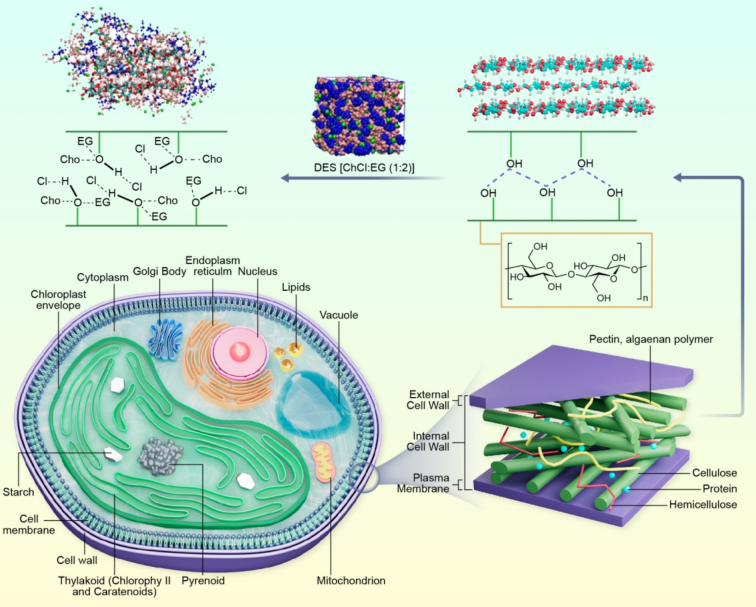
Mechanism of cell wall disruption of microalgae using ChCl:EG.

Because of these features, the use of DESs for the isolation of carotenoids from microbial biomass has increased rapidly. Recent reviews have highlighted the advancements of DESs and NADESs for extracting carotenoids from different biomass sources, exploring their evolution of key process variables, targeted compounds, extraction approaches, and prospects (Airouyuwa et al., 2024). Despite these developments, the use of DES in carotenoid isolation from microbial biomass remains in the early stage (Table 1). Most known DESs are polar and are primarily used for microbial biomass pretreatment with the additional addition of organic solvents. DES has the capability to lyse the microbial cell wall, where cellulose and polysaccharides are dissolved. These are the major structural elements that contribute to the mechanical strength of the microbial cell wall. For example, *C. pyrenoidosa,* a microalga that produces a carotenoid (lutein) composed of cellulose (15 %), glucosamine (3 %), hemicellulose (31 %), protein (27 %), lipid (9 %), and ash (5 %) (Northcote et al., 1958). Recently, Muhammad, Jahanbakhsh-Bonab, et al. (2024) studied the mechanism of cell disruption of *C. pyrenoidosa* using ChCl-based DES. Results revealed that cellulose chains were disrupted by DES species [choline (Ch), chloride (Cl^−^), and ethylene glycol (Eg)], and have lost their crystalline structure. Mechanism of disintegration *C. pyrenoidosa* is shown in Fig. 3.

**Table 1:** Pretreatment of microbial biomass using DESs for carotenoids recovery.

Microbial species	DES molar ratio	Optimum experimental conditions	Extraction yield carotenoids	Comparison with control	Reference
*C. pyrenoidosa*	ChCl:Eg (1:2)	Temperature (Temp) (45 °C), time (40 min), biomass to ethanol ratio (1:23.34 g/mL)	Lutein (3.80 mg/g)	__	(Zhang et al., 2025)
*C. pyrenoidosa* (80 % water content)	ChCl:Eg (1:2)	Temp (35 °C), time (40 min), biomass to ethanol ratio (1:23.34 g/mL)	Lutein (2.57 mg/g)	__	
*C. pyrenoidosa*	ChCl:Eg (1:2)	Pretreatment with DES for 5 min at room temp 28 °C, followed by extraction with 2-MTHF using vortex mixer for 30 s from freeze dried biomass	Lutein (118.14 mg/100 g)	31.66 mg/100 g untreated	(Muhammad, Jahanbakhsh-Bonab, et al., 2024)
*H. pluvialis* (Wet biomass)	ChCl:U (1:2)	Aqueous two-phase systems (ATPS) formed by 35 % DES, 30 % K_2_HPO_4,_ and H_2_O at pH 7.5, 50 °C, 1 h.	Astaxanthin (99 %)	__	(Nemani et al., 2024)
*C. pyrenoidosa*	ChCl:Gly (1:2)	Temp (40 °C), time (20 min), solid liquid ratio (1:33.3), solvent, *i.e.*, ethyl acetate (EA)	Lutein (1.35 mg/g)	a0.91 mg/g alkaline treatment	(Muhammad et al., 2022)
*D. salina*	ChCl:U (1:2)	DES was mixed with microalgae, temp 29 °C for 24 h with continuous stirring at 150 rpm.	Carotenoids (84 %)	62.3 % without pretreatment	(Asevedo et al., 2023)
*Schizochytrium* *sp.*	ChCl:AA (1:2)	Solvent to biomass ratio 20:1 *w*/w, time 1 h, temp 50 °C with ultrasonication	3.79 mg/g	__	(Ozel et al., 2024)
*C. vulgaris*	ChCl:AA (1:2)		25.51 mg/g	__	
*Scenedesmus protuberans*	ChCl:AA (1:2)		33.55 mg/g	__	
*Neochloris texensis*	ChCl:AA (1:2)		15.16 mg/g	__	
*Schizochytrium* *sp.*	ChCl:U (1:2)		3.79 mg/g	__	
*C. vulgaris*	ChCl:U (1:2)		25.14 mg/g	__	
*Neochloris texensis*	ChCl:U (1:2)		12.22 mg/g	__	

a Traditional method (alkaline hydrolysis, extraction with 2-MTHF solvent) was used for lutein extraction as a control.

For cell lysis, it's very important to select the DES which can efficiently destroy the cell wall while simultaneously persevere the bioactivity of carotenoids. Mostly till now, ChCl-based DESs have been employed with HBD like Eg, Gly, and U which have shown their capability in cell lysising in microalgae for *C. pyrenoidosa* (Muhammad, Jahanbakhsh-Bonab, et al., 2024; Zhang et al., 2025), *H. pluvialis* (Nemani et al., 2024), and *Dunaliella salina* (Asevedo et al., 2023) for recovery of carotenoids, as shown in Table 1. Low lutein content was noticed when acidic DES [ChCl:MA, ChCl:AA, and ChCl:formic acid (FA)] was employed for the pretreatment of *C. pyrenoidosa* for lutein recovery. Similarly, a poor carotenoid yield was observed for ChCl:OA for pretreatment of *D. salina* (Asevedo et al., 2023). This occurs because acidic conditions promote the undesired trans–cis isomerization of the carotenoids once they are liberated during cell disintegration, leading to degradation (Yusof et al., 2022). Therefore, these findings suggest that acidic DESs are not suitable for microbial biomass pretreatment, specially when carotenoids are aimed to recover.

Except for carotenoids degradation, carotenoids migrate towards DES resulting in the loss of carotenoids during extraction. Asevedo et al. (2023) noticed the decrease in carotenoids when *D. salina* was pretreated with DES. Authors compared the one-pot and two-step methods. One-pot method improved the carotenoid recovery up to 20 %. On the other hand, Men-based DES have been used to pretreat the microbial biomass, showing the capability to disrupt the cell wall of microalgae biomass, which improved the lutein content (0.86 mg/g) in comparison with the control (0.31 mg/g), which is around 177.42 % (Muhammad, Jahanbakhsh-Bonab, et al., 2024). Therefore, DESs are preferred because they have the capability to dissolve carotenoids, maintain their stability, and disrupt the cell wall of microbial biomass. In view of promising results, future work should focus on exploring new DESs as ChCl:EG, ChCl:Gly, ChCl:U and ChCl:AA are widely used in microbial biomass pretreatment for carotenoids recovery.

### 4.6 Recent advances of DESs for carotenoid extraction from microbial biomass

**Fig. 4:**
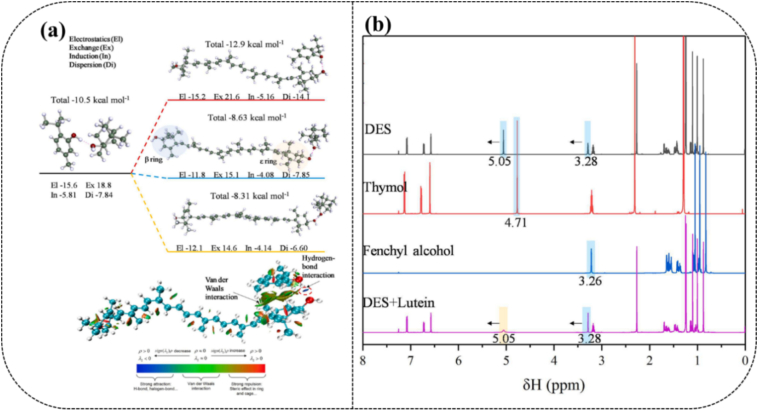
BE and physical components of BE calculated at SAPT2+/aug-cc-pVDZ level on the left-hand side (top), non-covalent interaction b/w lutein and thymol analysis (A) on left hand side bottom (a); ^1^HNMR chemical shifting FenAl + Thy before and after lutein extraction (b) (Fan et al., 2022).

The issue related to higher viscosity can be addressed by employing NADESs with lower viscosity and polarity, for instance, terpene-based NADESs, composed of Men and fatty acids (Yu et al., 2022). Al Fuhaid et al. (2024) compared eight different hydrophobic NADESs *i.e.*, Men:AA (1:1), Men:lactic acid:water (Wat) (3:3:1), Glu:Gly:Wat (1:1:4), Gly:citric acid (CitA):Wat (1:1:4), Glu:CitA:Wat (1:1:5), Men:caprylic acid (CA) (1:1), Bet:U:Wat (1:1:3), Glu:Gly:CitA:Wat (1:1:1:3) for astaxanthin recovery from engineered *Chlamydomonas reinhardtii*. The first, second and sixth NADESs demonstrated a higher capacity for astaxanthin extraction from dry biomass than traditional solvents such as acetone yielding 10.4, 12.8 and 13.4 mg/g, in contrast to 6.6 mg/g, respectively. Fan et al. (2022) designed the NADES system for fenchyl alcohol (FenAl)/thymol (Thy) with a molar ratio (1:1) for the effective extraction of lutein from microalgae biomass and found a higher lutein yield (4.3 mg/g) as compared to the conventional solvent (EA, *i.e.*, 3.90 mg/g) at 60 °C in 70 min. NADES improved lutein stability over high temperatures, light exposure, and prolonged storage conditions. Further mechanism of lutein extraction was studied *via* MD simulations. Fig. 4a shows the binding energy (BE) of hydrogen bond formation between FenAl:Thy NADES and lutein. It can be seen that the BE between thymol of NADES and lutein is −12.9 kcal/mol, and the BE between FenAl and Thy is −10.5 kcal/mol. This indicates that the H-bonding between Thy and lutein strongly influences the stability of targeted species such as lutein.

Noticeably, the electrostatic and dispersion interactions play a vital role in establishing precise H-bonding between the NADES and target species. In addition, the reduced density gradient (RDG) analysis (Fig. 4a downside), reveals that the VdW interaction between the ε-ionone ring of lutein and the aromatic ring of thymol also plays a crucial role in achieving enhanced recovery and stabilization. Notably, the active hydrogen of both components shifted to the higher field after NADES synthesis, particularly for thymol. Incorporation of lutein results in a peak broadening due to the existence of active hydrogen in thymol, suggesting stronger intermolecular interactions (Fig. 4b).

Rodrigues et al. (2020) identified Men/MyrA as the most efficient DES for astaxanthin extraction from crab shell waste. At the optimum condition (temp 60 °C, time 2 h) yield achieved was comparable to that obtained from 6 h Soxhlet extraction using conventional solvent acetone. Furthermore, the same approach was used for *H. pluvialis* biomass and mussels, resulting in yield increased from 3 to 657-times. Furthermore, Pitacco et al. (2022) explored the DESs prepared using terpenes (Men, Thy, and geraniol) and oleic acid (OlcA) for astaxanthin from freeze-dried *H. pluvialis* biomass and culture broth. Examined, DES achieved 60 % astaxanthin from freeze-dried biomass within 6 h, with stirring at ambient temperature, yield increasing up to 83 % when extraction was carried out for 24 h. When the same DES was applied directly to culture broth, it yielded only 30 % after 6 h and 68 % after 48 h, indicating that water in culture broth limits extraction efficiency (Pitacco et al., 2022). These findings suggest that HDESs could enhance extraction from untreated wet cells. Supporting this finding, Artigas-Hernández et al. (2023) demonstrated that pretreating *X. dendrorhous* cells with a pulsed-electric field (PEF), followed by aqueous incubation for 24 h prior to lyophilization, significantly improved astaxanthin extraction efficiency *via* Thy/salol (Sal) from 55 to 79 %. Thy/Sal outperformed other terpene-based DESs in extracting astaxanthin from both unpretreated and pretreated biomass (Artigas-Hernández et al., 2023). Table 2 summarises the application of various DESs for the extraction of carotenoids from microbial biomass. Based on the literature, thymol-based DESs were found to be the most efficient for the recovery of carotenoids (astaxanthin and lutein).

**Table 2:** Summary of the recent studies on the extraction of carotenoids using DES and NADES.

Source of the product	Extracting DES composition	Extraction method	Targeted compound yield	Comparison with control	Reference
*Scenedesmus sp*	FenAl:Thy (1:1)	Stirring at 1200 rpm and 30 °C for 70 min.	Lutein (6.26 mg/g)	3.91 mg/g	(Fan et al., 2022)
*H. pluvialis*	Thy:OlcA (1:1)	Stirring at 50–100 rpm and room temp for 6 h.	Astaxanthin (60 %)	a100% (Solvent mixtture)	(Pitacco et al., 2022)
*Xanthophyllomyces dendrorhous (yeast)*	Thy:Sal (0.3:1);	Pretreated with pulse electric field, time 24 h.	Astaxanthin (79 %)	b100% (DMSO)	(Artigas-Hernández et al., 2023)
*Paracoccus carotinifaciens*	ChCl:AA (1:3)	Time 8 min, extraction temp 65 °C, solid/liquid ratio 0.05 g/mL, amplitude level: 15 %.	Astaxanthin (1500 μg/mL)	150 μg/mL	(Paz et al., 2024)
*H. pluvialis*	ChCl:D-Fru	ATPS formed by ILs, DES, and H_2_O, solid to liquid ratio (1:20), temperature 35 °C and time 3 h.	Astaxanthin (2.35 mg/g)	1.96 mg/g	(Zhang et al., 2024)
*C. zofingien sis*	OctA:DecA (2.3:1)	(Ultrasonication at 50 °C for 49 min, and solid to solvent ratio 66.2 mg/mL.	Canthaxanthin (70.4 μg/mL extract)	50.9 μg/mL	(Yang et al., 2023)
*Haloferax mediterranei (wet biomass)*	Men:LeA (1:1)	Stirring, solid to liquid ratio (1:10), temperature 25 °C, and time 1.8 h.	Bacterioruberin (5.2 mg/g)	0.76 mg/g	(Kholany et al., 2023)
*C. vulgaris*	Nonanoic:DodA (1:1)	Solvent to sample ratio (27.33:1), time 33 min.	Lutein (1.42 mg/mL)	__	(Sportiello et al., 2024)

a Total astaxanthin content in *H. pluvialis* was determined using solvent mixture (cyclohexane/ethanol/acetone). As a control and for comparison this value was considered 100%.

b Total astaxanthin content in *H. pluvialis* was determined from bead-beaten biomass using dimethyl sulfoxide (DMSO) solvent. As a control and for comparison this value was considered 100%.

**Fig. 5:**
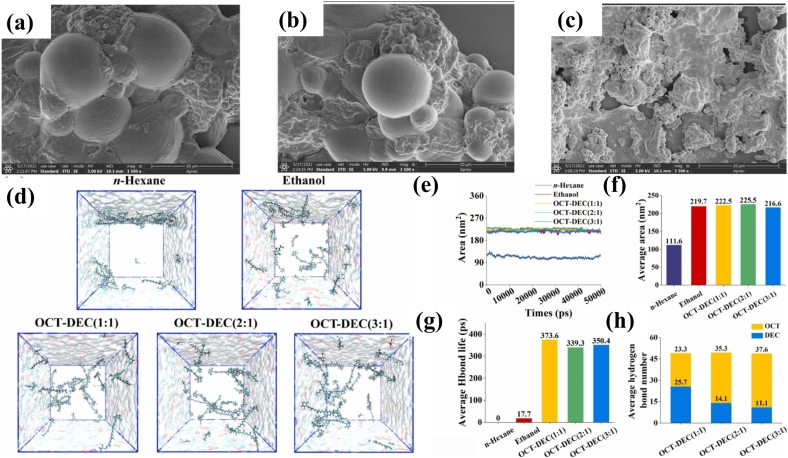
Morphology of *C. zofingiensis* cells before extraction (a); extraction using n-hexane (b); extraction with OctA-DecA (c); MD simulations analysis: images of canthaxanthin dissolved in (n-hexane, ethanol, and OctA-DecA) at 0 ps and 50,000 ps (d); SASA (e); average SASA (f); average lifetime of hydrogen bond (g); and average hydrogen bond number (h) between canthaxanthin molecules and various solvents (Yang et al., 2023).

Canthaxanthin is a ketocarotenoid like astaxanthin that was effectively extracted from lyophilized *C. zofingiensis* using HDESs (Yang et al., 2023). Seven HDESs were examined and exhibited comparable or superior extraction efficiency to ethanol and n-hexane. Under the optimized conditions, octanoic acid (OctA)/decanoic acid (DecA) yielded 70.4 μg/mL of canthaxanthin from unmilled dry biomass, surpassing 62.2 μg m/L obtained from grinded biomass with ethanol. The results indicate that OctA/DecA not only facilitated cell lysis but also improved the extraction yield (Yang et al., 2023), as shown in Fig. 5. During the extraction of the conventional solvents most of the cells were found smooth and in contact with each other (Fig. 5b). On the contrary, the cell wall was entirely disrupted after OctA-DecA treatment (Fig. 5c), demonstrating its strong penetration and erosion caused by ultrasound and solvent. This observation confirms the hypothesis that OctA-DecA has the capability to disrupt the cell wall.

Several studies have used the HDES for lipid-soluble compounds, but limited information is available on the mechanism of dissolution for the desired compounds. HBD formation between the ESs and the targeted components is important to enhance extraction efficiency. Yang et al. (2023) carried out the MD simulations to investigate HBDs formation in the OctA-DecA system and the behaviour of canthaxanthin molecules. Results revealed that canthaxanthin monomers aggregated and clusters formed while using the conventional solvents (hexane or ethanol) during the simulation period of 50,000 ps (Fig. 5d**)**. In contrast, using OctA-DecA (2:1) monomers were distributed uniformly, showing the high solubility of canthaxanthin (Fig. 5d). The solvent accessible surface area (SASA) did not change remained stable for all solvents except hexane (Fig. 5e**)**, aligning with the cluster development of canthaxanthin in n-hexane while uniform distribution observed in OctA-DecA (Fig. 5d**)**. The average SASA for canthaxanthin was more (225.5 nm^2^) in Oct-Dec (2:1), indicating greater stability compared to other solvents (Fig. 5f**)**. The average hydrogen bond lifetime in OctA-DecA ranges from 339.3 to 373.6 ps, significantly longer than the 17.7 ps in ethanol, suggesting that ethanol forms less stable bonds with canthaxanthin (Fig. 5g**)**. Additionally, OctA-DecA (2:1) forms an average of 49.4 HBDs with canthaxanthin, surpassing the counts for other ratios **(**Fig. 5h**).**

The extraction of β-carotene using DES has been explored less from microbial biomass. While various studies have been reported on the use of DESs for extracting β-carotene from agricultural waste (pumpkin), vegetables (Stupar et al., 2021), and orange peels (Viñas-Ospino, Panić, et al., 2023). Viñas-Ospino, Panić, et al. (2023) conducted the study on the isolation of β-carotene from orange peels using nine HPLDESs and HDESs. Results indicated that β-carotene has a high solubility in HDESs, especially L-Men/eucalyptol (Euc) (1:1), 30 min, room temperature, and shaking at 150 rpm yielded (168.7 mg/100 g). This can be explained by Men-based DES having a lower viscosity than other DES, but a higher viscosity than conventional solvents. Another study found that among the ten HDESs, CA/capric acid (3:1) integration with UAE exhibited the best efficacy for β-carotene extraction from pumpkin (Stupar et al., 2021). In light of these supporting results, exploring the use of DES for β-carotene extraction from microbials is warranted. Although the processing methods for microbial biomass differ from those of plant-materials because of their distinct cellular structure and biochemical composition, the above-mentioned efficiency of HDESs in dissolving and stabilizing β-carotene suggests potential for successful applications in this area.

## 5 Challenges, perspectives, and implementation of DES-based carotenoids extraction

Although promising results have been achieved in the literature, DES-based carotenoids extraction from microbial biomass is still in the experimental proof of concept stage where lab-scale experiments have been performed, pilot scale processes are yet to be established. To make the microbial biomass pretreatment and extraction of carotenoids process feasible at a commercial scale using DES, there are various challenges that should be overcome; the first one is viscosity, which can limit mass transfer during extraction, resulting in longer extraction times and reduced efficiency. Optimization of the viscosity of DES plays a vital role in improving extraction efficiency (Zou et al., 2023). For example: Zhang et al. (2024) demonstrated the extraction of astaxanthin from shrimp shells using ChCl:1,2-butanediol (1:5). Where 0–20 % water was added to DES to reduce the viscosity, and astaxanthin recovery was improved at 10 % concentration of water by 2.43 times than DES. In another study, methanol was used as a co-solvent with DES. ChCl:tartaric acid (TA) (2:1) and methanol were used to extract carotenoids from apricot by-product waste *via* microwave-assisted extraction method (MAE). MAE-ChCl:TA-methanol (80:20) improved the extraction yield (76.11 mg/100 g) as compared to the conventional solvent (26.5 mg/100 g) (Koutsoukos et al., 2019). The second one is DES non-volatility, making them difficult to remove by evaporation, and they may need another washing step when drying leftover biomass for valorization. The third one is DES preparation and recycling methods, which involve several steps that can contribute to high operational costs. The industrial-scale economic viability assessment of DES will provide confidence for its application on a commercial scale-up (Isci & Martin, 2022).

It's important while developing a process to make a high-level evaluation of the solvent cost, impact, synthesis cost of the solvent and feed stock nature. Considering these factors into account will help you choose the best solvents for process. Furthermore, the tools like techno-economic analysis (TEA) provide valuable insights for more detailed estimations. These assessments must be conducted using cradle to grave methodology (Clarke et al., 2018).

### 5.1 Sustainability assessment of DES-based carotenoids extraction

Techno-economic analysis (TEA) is a valuable technique for assessing the feasibility of a proposed process, recognizing the issues and bottlenecks that hinder economic and technical viability at an early stage of research. It usually identifies the dominant factors that determine production cost. TEA normally includes capital costs, operational costs, and minimum selling price of the product by conducting energy and mass balances using simulations according to experimental data (Zhao et al., 2022).

The solvent cost is an important problem when DES is proposed for industrial applications as an alternative solvent. Mainly in the synthesis of DES, cost is governed by the components. Viñas-Ospino, Panić, et al. (2023) reported the cost estimation of DodA/OctA (1:3), L-Men/Euc (1:1), and L-Men: DL-Cam (1:1) cost 58, 184 and 140 €/kg. However, the first solvent was cheap but showed higher degradation of carotenoids. In contrast, the third showed better extraction efficiency, stability, and lower cost than the second. Recently, Rente et al. (2022) performed an economic assessment of traditional and DES-assisted processes under identical conditions (mixing at room temp for 2 h), but downstream processing differed for anthocyanins. The traditional extraction process yielded crude anthocyanins after solvent removal. In the DES procedure, two scenarios were considered: (i) direct use of the extract and (ii) purification (adsorption chromatography or crude extract). On economic evaluation, it was found that scenario 1 has the lowest capital (CAP) investment of 450,000€ and annual operating (AO) cost of 170,000€ compared to scenario 2 (657,000€, 240,000€), where a complex DES removal approach is required before further use of extract, and traditional CAP (570,000€), AO cost (190,000€) for 50 L-scale. In addition, synthesis of the DES was also found to be the main contributor to energy consumption when Life Cycle Assessment (LCA) of a DES-based-hydrothermal process was conducted (Song et al., 2023). Therefore, an effective method for recovery of the DESs is required to reduce the raw material and operation cost (Kumar et al., 2020), and more studies are required for ready to use extracts as this approach has a positive impact on the cost of the process as well as on sustainability (Rente et al., 2022).

Besides TEA, the environmental impact of a process is a crucial factor to be addressed. LCA is an effective tool for evaluating the potential environmental impacts of a process during its entire life cycle, by using an input and output approach. For example, Zaib et al. (2022) evaluated the environmental impact of reline DES (ChCl/U), which was based on its production. They conducted LCA of its application (as a chemical reaction medium) and compared it with traditional solvents, including ethanol, EA, methanol, and dichloromethane (DCM)]. The findings indicated that DES showed less environmental impacts than both DCM and EA. In the context of biorefinery processes, other factors, like food grade status of the solvent and other valuable effects (enhanced bioactivity and bioavailability) of the extracted compound, must be considered. Furthermore, Zaib et al. (2022) compared the environmental performance of reline with other DES based on ChCl using different HBDs, such as CitA. They suggested that ChCl with CitA had a more environmental impact as a result of citric acid production by fermentation. However, there are limited published articles addressing application of LCA to extract carotenoids *via* DES. A study conducted by Martins et al. (2021), where LCA was conducted to analyse the environmental impacts of the proposed extraction and fractionation of pigments (chlorophyll and fucoxanthin) from *Saccharina latissimi via* ILs. The results showed that the primary contributors to environmental impacts were fossil resource depletion and electricity consumption. Notably, reusing ILs led to a significant 8–14 % reduction in overall environmental impacts, highlighting the benefits of sustainable practices in this process. Furthermore, research is warranted to compare the extraction of carotenoids using DESs with other documented approaches in the literature. For example, Kyriakopoulou et al. (2015) conducted a comparison between conventional and innovative extraction approaches (microwave and ultrasound) to intensify the process.

Overall, a comprehensive strategy must be formulated to encourage the use of DESs for industrial applications in carotenoids extraction from microbial biomass, encompassing all considerations such as sustainability, scalability, economic viability, solvent recovery, and reusability without a readily usable extract. For that, TEA and LCA are holistic tools for evaluating a case study process. Each tool independently identifies the most impactful actions, but when combined, they maximize decision-making potential from both economic and environmental perspectives.

### 5.2 Technological challenges

Implementation of DES for microbial processing of carotenoids still faces some challenges. One of the main issues is the cost of DESs, though many studies have claimed that DESs are cost-effective. For the pretreatment of microbial biomass and extraction of carotenoids using DESs, a high DES to biomass ratio is required *i.e.*, 20:1 or 50:1 (Fan et al., 2022; Pan et al., 2017). A huge amount of DES is consumed, resulting in high cost and making it difficult to scale up carotenoid production from microbial biomass. Meanwhile, many researchers are trying to reduce synthesis and purification costs, improve recovery and explore low-cost different approaches for processing microbial biomass. Although the DES are recyclable, their effectiveness in biomass processing decreases over time. This reduced efficiency in successive cycles may be due to the presence of impurities during the cycles while the DES is being recycled (Muhammad, Jahanbakhsh-Bonab, et al., 2024). DES loss during the biomass processing makes the process complicate and increases the cost.

DES recovery from the pretreated biomass is important because it reduces the cost, as recycling them from the pretreated biomass aligns with the economic viability (Ferrari et al., 2021). Most of the reports in the literature have focused on the recovery of DES, which are discussed here. In most studies where DES has been used for microbial biomass pretreatment, solid-liquid separation (centrifugation) has been used without any additional solvent. However, few studies are addressing the recovery rate and reuse performance of DES in microbial biomass pretreatment. Muhammad, Jahanbakhsh-Bonab, et al. (2024) was able to recover 80 % of the solvent by pretreating the microalgae biomass. Unfortunately, further recycling the DES till the fourth cycle recovery as well as the extraction efficiency decreased. Despite this technique, DES was recovered *via* membrane filtration and recrystallization methods for microbial biomass disintegration. Membrane filtration was found to be a more energy-consuming method, which escalated the whole energy of the process (Song et al., 2023).

Besides this, carotenoids are also recovered from the biomass without pretreatment using DES as discussed in Section 4.6. A number of approaches, including liquid-liquid extraction, antisolvents, solid-phase extraction (SPE), and chromatography, have been discussed for compounds recovery from DES/NADES (Görüşük et al., 2024). Nam et al. (2015) investigated the recovery efficiency of SPE and antisolvent methods. The recovery efficiency of the DES *via* the SPE method was higher (92 %) than the anti-solvent (75 %). Recovery of DES was successfully performed *via* a C18 SPE cartridge loaded with coconut-based activated carbon. The SPE method was preferred because of its cost-effectiveness, convenience, and speed. Recently, Görüşük et al. (2024) highlighted the recovery efficiency of carotenoids from DES/NADES along with the reuse of DES and other materials (microporous resin) to enhance the sustainability of the separation process. DES, tributyl phosphate (TBP):AA(1:2) recycled three times, and extraction efficiencies were found to be 90 % (± 3.2 %), 83 % (± 2.9 %), and 75 % (± 3.5 %), respectively, showing that DES can be effectively recycled, maintaining reasonable carotenoid yield. Regarding the recyclability and isolation of carotenoids from microbial biomass *via* DES/NADES are limited which is evident of the significant gap in the literature. DES, a “new” class of solvents, has received less research attention and exploration, limiting our understanding of their characteristics and carotenoid interactions. Moreover, DESs are more biocompatible than ILs. Some studies proposed the direct use of DES extracts without purification (Rente et al., 2022).

## 6 Conclusion

Natural carotenoids from microbial biomass offer safer and alternative sources of synthetic carotenoids, providing sustainability and versatility. However, the current downstream process for microbial carotenoid production involves hazardous solvents and energy-consuming processes with low yield. DES-based extraction is a revolutionary approach that marks a significant transition to more sustainable and efficient extraction processes, particularly for carotenoid extraction. Due to the increasing demand for green technologies to extract valuable compounds from microbial biomass, DES offers several benefits, including eco-friendliness, cost-efficiency, and biodegradability. DES-extracted carotenoids possess improved solubility, stability, and bioavailability. Despite this, there are still several challenges related to these solvents: high cost, high viscosity, difficult to recycle. Additionally, the environmental impact of carotenoid extraction and processing using DES remains unclear. Because of these reasons, future directions are suggested: 1) Further optimization of the process, DES composition can enhance the efficiency of the extraction process and can reduce the cost; 2) developing a more efficient approach for the recovery of DES, isolation of the carotenoids from the DES and direct use of the carotenoids extracts; 3) perform the LCA to evaluate the environmental impact. By overcoming these obstacles, we believe that continuous focus on the DES in microbial biomass processing will ultimately displace conventional solvents on an industrial scale and achieve sustainable development goals.

## CRediT authorship contribution statement

**Gul Muhammad:** Writing – original draft, Funding acquisition, Data curation, Conceptualization. **Zhenglong Li:** Writing – review & editing. **Cheng-Ye Ma:** Software. **Qiong Wang:** Writing – review & editing. **Ximing Zhang:** Writing – review & editing, Supervision, Conceptualization.

## Declaration of competing interest

The authors declare that they have no known competing financial interests or personal relationships that could have appeared to influence the work reported in this paper.

## Data Availability

Data will be made available on request.
